# Advanced Computational Methods for Oncological Image Analysis

**DOI:** 10.3390/jimaging7110237

**Published:** 2021-11-12

**Authors:** Leonardo Rundo, Carmelo Militello, Vincenzo Conti, Fulvio Zaccagna, Changhee Han

**Affiliations:** 1 Department of Radiology, University of Cambridge, Cambridge CB2 0QQ, UK; 2 Department of Information and Electrical Engineering and Applied Mathematics (DIEM), University of Salerno, 84084 Fisciano, Italy; 3 Institute of Molecular Bioimaging and Physiology, Italian National Research Council (IBFM-CNR), 90015 Cefalù, Italy; 4 Faculty of Engineering and Architecture, University of Enna KORE, 94100 Enna, Italy; vincenzo.conti@unikore.it; 5 Department of Biomedical and Neuromotor Sciences, University of Bologna, 40138 Bologna, Italy; fulvio.zaccagna@unibo.it; 6 IRCCS Istituto delle Scienze Neurologiche di Bologna, Functional and Molecular Neuroimaging Unit, 40139 Bologna, Italy; 7 Saitama Prefectural University, Saitama 343-8540, Japan; mykallis13@gmail.com

The Special Issue “Advanced Computational Methods for Oncological Image Analysis”, published for the *Journal of Imaging*, covered original research papers about state-of-the-art and novel algorithms and methodologies, as well as applications of computational methods for oncological image analysis, ranging from radiogenomics to deep learning. Interesting review articles were also considered.

Nowadays, the amount of heterogeneous biomedical data is constantly increasing, owing to the advances in image acquisition modalities and high-throughput technologies [1,2,3]. In particular, this trend applies to oncological image analysis [4]. Cancer is the second most common cause of death worldwide and encompasses highly variable clinical and biological scenarios. Some of the current clinical challenges are (i) early disease diagnosis and (ii) precision medicine, which allows for treatments targeted at specific clinical cases. The ultimate goal is to optimize the clinical workflow by combining accurate diagnosis with the most suitable therapies [5].

The automated analysis of these large-scale datasets creates new compelling challenges that require advanced computational methods, ranging from classic machine learning techniques [6,7] to deep learning [8,9].

The developed reliable computer-assisted methods (i.e., artificial intelligence), together with clinicians’ unique knowledge, can be used to properly handle typical issues in evaluation/quantification procedures (i.e., operator dependence and time-consuming tasks) [10]. These technological advances can significantly improve result repeatability in disease diagnosis and act as a guide towards appropriate cancer care. Indeed, the need for applying machine learning and computational intelligence techniques to effectively perform image processing operations ―such as segmentation, co-registration, classification, and dimensionality reduction, and multi-omics data integration―has steadily increased.

This Special Issue collects 13 papers related to oncological image analysis, including 10 original contributions and 3 review articles.

In the last few years, the role of medical image computing and quantification has been remarkably growing. Several areas have benefited from these advances, including oncology, since the advancement of computational techniques provides a technological bridge between radiology and oncology. This aspect could significantly accelerate the adoption of precision medicine. Regarding medical imaging focusing on traditional image analysis tasks―such as registration, fusion, and segmentation―in recent years we have witnessed the advances of model-based medical image processing for biomarker development [11].

Among sex-related cancers, breast cancer for women and prostate cancer for men are major causes of disease and death.

Concerning breast cancer, methods to predict its risk or to stratify women in different risk levels could help achieve early diagnosis and consequently, mortality reduction. Literature reviews are useful in providing a comprehensive vision of computer-assisted approaches to support the clinical process, especially for young scientists [12,13]. In particular, [14] reviews extraction methods of textural features from mammograms, where machine learning and deep learning algorithms are used to infer knowledge from the features and assess breast cancer risk. The accurate diagnosis of breast cancer is very challenging due to the increasing disease complexity, such as changes in treatment procedures and patient population samples. Improving the performance with suitable diagnosis techniques could lead to personalized care and treatment, thus reducing and controlling cancer recurrence [15].

Even though magnetic resonance (MR) has a better capability to differentiate soft tissues, mammography is the primary imaging modality used for the screening and early detection of breast cancer. The analysis of mammography images starts with detecting regions of interest around tumors. Those regions are then delimited through segmentation and classified as probably benign or malignant tumors. Meanwhile, the manual detection and delimitation of masses in images is time consuming and error prone. Therefore, integrated computer-aided detection systems have been proposed to assist radiologists in the process [16].

Along with the well-known imaging modalities, such as MR, CT, PET, US, which are now consolidated and used in clinical routine, recently new modalities have emerged that exploit techniques initially born in non-clinical contexts, such as microwaves [17,18]. When the aim is to reconstruct the dielectric/conductivity profile of the tissue under examination, “quantitative” algorithms must be adopted. In these cases, the reconstructions are basically optimized iteratively to consider the non-linearity. Among linear imaging methods, commonly addressed as radar approaches, beam forming (BF) is probably the most popular in microwave breast imaging. Basically, it consists of time-shifting the signals received over the measurement aperture to isolate signals scattered from (and hence to focus at) a particular synthetic focal point belonging to the imaged spatial area [17]. Microwave-based tomography is a model-based imaging modality that approximately reconstructs the actual internal spatial distribution of a breast's dielectric properties over a reconstruction model consisting of discrete elements. Breast tissue types are characterized by their dielectric properties, so the complex permittivity profile could help distinguish different tissue types [18].

Prostate cancer is one of the most diagnosed cancers in men and can often cause bone metastases. In this case, the most common imaging technique for screening, diagnosis, and the follow-up of disease evolution is bone scintigraphy, due to its high sensitivity and widespread availability in nuclear medicine facilities. To date, the assessment of bone scans relies solely on the interpretation of an expert physician who visually assesses the scan. This time-consuming task is also subjective, due to the lack of well-established criteria to identify bone metastases and quantify them using a straightforward and universally accepted procedure. The aim of the work in [19] was to provide the physician with a fast, precise, and reliable tool to quantify bone scans and evaluate disease progression/response to treatment.

Immunotherapy is one of the most significant breakthroughs in cancer treatment. Unfortunately, only a few patients respond positively to the treatment. Moreover, to date, no efficient biomarkers exist for discriminating patients eligible for this treatment in an early stage. To help overcome these limitations, the development of tools for discriminating between patients with high chances of response and those with disease progression is needed [20].

Among tumors, brain lesions are one of the foremost reasons for the rise in mortality among children and adults. A brain tumor is a mass of tissue that propagates out of control of the normal forces that regulate growth inside the brain [21]. The quantitative analysis of brain tumors provides valuable information for understanding tumor characteristics and planning better treatment. The manual segmentation of brain tumors is a challenging and time-consuming task. The accurate segmentation of lesions requires multiple image modalities with varying contrasts. As a result, manual segmentation, which is arguably the most accurate segmentation method, would be impractical for more extensive studies. Moreover, automated brain tumor classification on MRI is non-invasive, so that it avoids biopsy and makes the diagnosis process safer. The effort of the research community to propose automatic brain tumor segmentation and classification methods has been tremendous. As a result, ample literature exists on segmentation using region growing, traditional machine learning and deep learning methods [22,23]. Similarly, a number of tasks have been successfully conducted in the area of brain tumor classification into their respective histological type.

Structural and metabolic imaging are fundamental for diagnosis, treatment and follow-up in oncology. Beyond the well-established diagnostic imaging applications, ultrasounds are currently emerging in clinical practice as a non-invasive technology for therapy. Indeed, the sound waves can increase the temperature inside the target solid tumors, leading to the apoptosis or necrosis of neoplastic tissues. The MR-guided focused ultrasound surgery (MRgFUS) technology represents a valid application of this ultrasound property, mainly used in oncology and neurology [24]. Patient safety during MRgFUS treatments was investigated because temperature increases during the treatment are not always accurately detected by MRI-based referenceless thermometry methods. For these reasons, in-depth studies about these aspects are needed to monitor temperature and improve safety during MRgFUS treatments.

Deep learning approaches represent state-of-the-art techniques in many clinical scenarios, allowing for excellent performance. In the clinical setting, the main problem derives from their black-box approach (i.e., the nature of neural networks)—understanding and interpreting their internal mechanisms are difficult. Moreover, they require a training phase on large-scale datasets. These drawbacks undermine their immediate clinical feasibility. Apart from that, deep learning architectures, specifically convolutional neural networks (CNNs), are well-established in image analysis, processing, and representation. They can optimize feature design tasks that are essential to automatically analyze different types of medical images [25,26,27]. Various approaches have been developed using CNN architectures, aiming to support the clinical routine, such as tumor segmentation [16], skin melanoma prediction [28], and the estimation of the immunotherapy treatment response [20].
